# Ventral Spinal Epidural Hematoma Triggered by Self-Urinary Catheterization and the Role of Diffusion-Weighted Imaging in Early Detection

**DOI:** 10.7759/cureus.78445

**Published:** 2025-02-03

**Authors:** Kae Ishii, Yasutaka Murakami, Keisuke Kitamura, Hideaki Kanki

**Affiliations:** 1 Department of Neurology, Osaka Police Hospital, Osaka, JPN

**Keywords:** back pain, diffusion-weighted imaging, hemorrhage, magnetic resonance imaging, spinal epidural hematoma

## Abstract

Spontaneous spinal epidural hematomas (SSEHs) are extremely rare and clinically recognized by sudden onset of severe neck pain and progressive neurological deficits. Ventral SSEH is particularly uncommon, accounting for less than 10% of SSEH cases. Early and accurate diagnosis is crucial, especially using magnetic resonance imaging (MRI). However, conventional MRI sequences occasionally exhibit unclear signal changes during the early stages of hemorrhage, which may be overlooked by clinicians unfamiliar with this uncommon disease.

This report presents the case of an 81-year-old man who developed a cervical ventral SSEH during self-urinary catheterization. Diffusion-weighted imaging (DWI) was instrumental in identifying the hematoma, as it revealed a high signal ventral to the spinal cord, which was not immediately apparent on conventional MRI sequences. The patient experienced acute posterior cervical pain and neurological deficits, including lower extremity paralysis, which spontaneously resolved within hours. Follow-up imaging demonstrated the resolution of spinal cord compression. This case underscores the diagnostic value of DWI in identifying acute ventral spinal epidural hematomas, particularly when conventional imaging modalities fail to provide definitive results.

## Introduction

Spinal epidural hematoma is a rare condition characterized by the sudden onset of severe pain followed by progressive neurological deficits. Spontaneous spinal epidural hematoma (SSEH) is defined as a condition with no known cause and accounts for approximately half of all cases [1]. Ventral SSEHs are rare, accounting for <10% of all SSEH cases [2]. Early diagnosis using magnetic resonance imaging (MRI) is crucial. However, conventional MRI sequences exhibit relatively low sensitivity to magnetic susceptibility effects, particularly during the early stages of hemorrhage. We report a case of SSEH in which diffusion-weighted imaging (DWI) was effective for the diagnosis.

## Case presentation

An 81-year-old man presented with acute posterior cervical pain during self-catheterization for urinary retention secondary to prostate cancer in October 2023. The patient was on levetiracetam and perampanel for epilepsy but had no history of trauma, hypertension, or antithrombotic therapy. On arrival, 40 min after symptom onset, his blood pressure was 208/100 mmHg, pulse was 94 beats/min, respiratory rate was 21 breaths/min, SpO2 was 97% on room air, and body temperature was 36.3°C. Physical examination revealed clear lung sounds, normal heart sounds, and absence of murmurs. However, even minimal movement of the neck exacerbated the pain. He was alert and had a Glasgow Coma Scale score of E4V5M6. The results of cranial nerve, motor, and sensory examinations were unremarkable.

**Figure 1:**
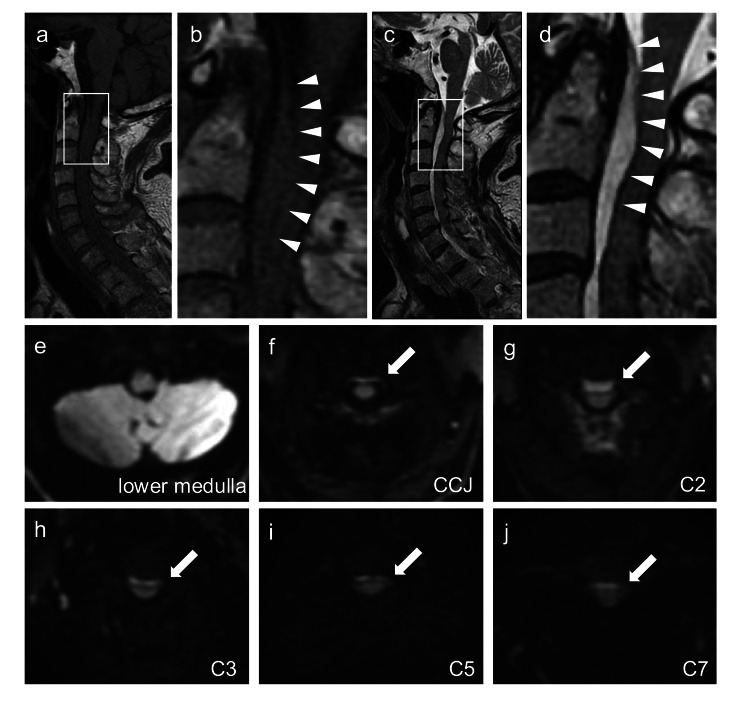
Spine magnetic resonance imaging findings 90 minutes after arrival a, b: Sagittal T1-weighted images; c, d: Sagittal T2-weighted images; e-j: Axial diffusion-weighted images (DWI; b=650 s/mm2) at the lower medulla, craniocervical junction, C2, C3, C5, and C7 levels. Enlarged views of the regions outlined by squares in panels a and c are shown in panels b and d, respectively. Arrowheads indicate a hematoma located ventral to the spinal cord, with posterior compression of the spinal cord observed near the C2 level. DWI shows no abnormality at the lower medullary level but reveals a hyperintense signal, consistent with a hematoma ventral to the cervical cord (arrows). The high-intensity signal is most prominent at the C2 level and extends from the craniocervical junction to the C7 level.

Computed tomography of the head and neck revealed no abnormalities. Shortly thereafter, the patient developed left lower extremity paralysis, with muscle strength reduced to Manual Muscle Testing (MMT) grade 1. MRI of the cervical spine performed 90 min post-admission using a 1.5 Tesla superconducting magnet (Signa HDxt 1.5T, General Electric Healthcare, Chicago, USA) revealed an isointense mass in the epidural space ventral to the cord on sagittal T1-weighted images. T2-weighted images demonstrated a hyperintense region from the craniocervical junction to the C7 level, with signal intensity slightly higher than that of the cerebrospinal fluid (Figure 1A-1D). DWI (b=650 s/mm2) revealed a high signal ventral to the cord along the same region, with no signal change ventral to the lower medulla (Figure 1E-1J). Based on the clinical course and imaging findings, cervical ventral spinal epidural hematoma was diagnosed.

After MRI, the MMT grade of both lower extremities further declined to 0, with reduced warmth and pain sensations below the T10 level. Approximately 30 min later, his muscle strength in both lower extremities improved to MMT grade 4. The next morning, his neurological deficits had nearly resolved.

**Figure 2:**
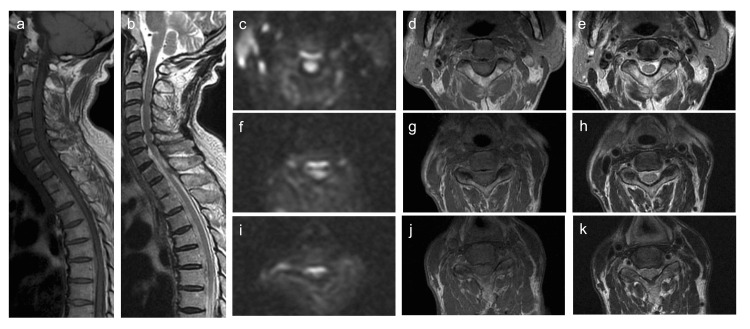
Spine magnetic resonance imaging findings on day 2 a: Sagittal T1-weighted image; b: Sagittal T2-weighted image; c, f, i: Axial diffusion-weighted images (DWI; b=650 s/mm2); d, g, j: Axial T1-weighted images; e, h, k: Axial T2-weighted images; c-e: At the C2 level; f-h: At the C5 level; i-k: At the C7 level. The hematoma spread thinly to the level of the lower thoracic spinal cord, with improved compression of the cervical spinal cord at the C2 level. DWI more clearly demonstrates the hematoma ventral to the spinal cord than axial T1- and T2-weighted images.

Follow-up MRI performed the day after admission showed resolution of the compression at the C2 level and thin extension of the hematoma from the craniocervical junction to the lower thoracic cord (Figure 2). DWI clearly demonstrated a hematoma on the ventral side of the spinal cord (Figure 2). Angiography showed no vascular abnormalities, and bone scintigraphy showed no metastasis. The final diagnosis was an SSEH. As the symptoms improved spontaneously, neither medication nor surgical intervention was necessary. The patient was discharged on the 11th day with a modified Rankin Scale score of 1.​​​​​

## Discussion

Here, we report a case of an SSEH that occurred during self-urinary catheterization. Conventional MRI sequences show limited signal changes, whereas DWI reveals high signal intensity ventral to the spinal cord, offering diagnostic clues.

Approximately 20% of SSEHs occur in the context of anticoagulant therapy, indicating a potential association between SSEHs and antithrombotic drugs [3,4]. However, no antithrombotic therapy was administered in this case. It is hypothesized that the hemorrhage was triggered by increased intra-abdominal pressure resulting from the self-urinary catheterization procedure. SSEH is thought to rupture the internal vertebral venous plexus (IVVP) located in the epidural space [5]. The IVVP lacks venous valves and is vulnerable to changes in venous pressure [6]. The development of SSEH during weight training, which increases intra-abdominal pressure, has been reported [7]. Ventral SSEHs are uncommon, and their rarity can be explained by two anatomical factors. First, the IVVP is developed dorsally. Second, the ventral IVVP is partially covered by the posterior longitudinal ligament, which separates it from the epidural space [5,8]. This rarity increases the risk of ventral SSEH being overlooked in clinical practice. 

The high signal intensity on DWI in the ventral spinal cord was important for diagnosis in this case. The signal characteristics of a hematoma on MRI change over time. Within the first 24 h, a hematoma typically appears isointense on T1-weighted images and slightly hyperintense on T2-weighted images [9]. In our case, it was challenging to differentiate acute hematomas from those of the cerebrospinal fluid and spinal cord because of the minimal signal difference. As noted by Iwatsuki et al., echo-planar gradient-echo T2*-weighted MRI is useful for assessing hemorrhage [10]. We propose the use of DWI to differentiate acute spinal epidural hematomas. Acute hematomas exhibit high signal intensity on DWI [11], clearly distinguishing them from other structures in SSEH cases. Additionally, DWI can help differentiate spinal epidural hematoma from spinal infarction, which may present with similar neurological symptoms [12]. Therefore, spinal DWI may be beneficial in cases where acute spinal cord lesions are suspected.

In this case, the rapid improvement of neurological symptoms was attributed to the hematoma’s redistribution within the epidural space, relieving spinal cord compression as confirmed by follow-up MRI showing the thinning and spreading of the hematoma. Previous reports indicated that 73-84% of patients who are managed conservatively achieve favorable outcomes [13,14]. Conversely, surgical decompression, reported in approximately 70% of cases, is often performed in cases of severe neurological deficits or when rapid clinical improvement is absent. Among patients initially treated conservatively, 9.5% of them eventually require operation because of clinical deterioration at an average of 7.5 days [4]. Therefore, even with conservative treatment, careful observation during the first week is necessary.

## Conclusions

We report a rare ventral spinal epidural hematoma triggered by self-urinary catheterization, highlighting the diagnostic challenges of this uncommon location and the critical role of DWI in early detection. This case highlights the potential for spontaneous recovery with conservative management and the value of DWI in differentiating acute spinal cord lesions.
